# Supplementary material for: In Vitro Propagation and Genetic Uniformity Assessment of Manglietiastrum sinicum: A Critically Endangered Magnoliaceae Species
Source: Plants (Basel). 2023 Jun 30;12(13):2500. doi: 10.3390/plants12132500 (PMC10346917; doi:10.3390/plants12132500)
Supplement: Supplementary file 1 [file plants-12-02500-s001.zip › plants-2423960-supplementary.pdf]

Pre- experiments of proliferation.

In the bud induction experiments, MS+0.5 mg/L BA + 0.05 mg/L IBA and MS+1.0mg/L BA +0.1 mg/L IBA were the relatively good medium for induction (the induction rate were both above 90%). While the induction rate was below 90% when the concentration of BA and IBA were higher. Since there was no design experiment between the concentrations of BA (0-0.5 mg/L, 0.5-1.0 mg/L) and IBA (0-0.05mg/L, 0.05-0.1mg/L) before. So we set pre- experiments of proliferation, in order to induce more adventitious buds on bud proliferation.

Methods. After induction, the buds were transferred into proliferation media. MS was supplemented with different concentrations of BA (0.2, 0.5, 0.8, 1.0 mg/L) in combination with IBA (0.02, 0.05, 0.08, 0.1 mg/L). Each treatment had three replications with a total of 30 explants. After 30 days, the proliferation rate and number of new buds per explant (≥0.5 cm) were recorded.

Results. After 30 days of proliferation, the proliferation effect was significantly different. We found the proliferation of the combination of 0.8 mg/L BA and 0.08 mg/L IBA was the best, the proliferation rate and average number of buds were high, and the buds were green and robust (Table S1).

Table S1. Pre-experiments of proliferation: Effect of different compositions and concentrations of BA and IBA on bud proliferation.

**Medium**	**Proliferation rate (%)**	**the number of buds per explant（length ≥ 0.5 cm）**
MS+0.2 BA+0.02 IBA	174.00±0.08	0.68±0.02
MS+0.5 BA+0.05 IBA	226.00±5.29	1.03±0.02
MS+0.8 BA+0.08 IBA	257.31±5.37	2.50±0.15
MS+1.0 BA+0.10 IBA	243.30±5.51	1.34±0.02
